# Biopolymers in Facial Aesthetics: Gel-Based Applications, Safety, Effectiveness, and Future Prospects—A Systematic Review of the Literature

**DOI:** 10.3390/gels11060455

**Published:** 2025-06-13

**Authors:** Gonzalo Ruiz-de-León, Daniela Cortés-Eslava, Esther Hernández-Pacheco, María-Ángeles Serrera-Figallo, Daniel Torres-Lagares, María Baus-Domínguez

**Affiliations:** Department of Stomatology, Faculty of Dentistry, University of Seville, C/Avicena S/N, 41009 Seville, Spain; ruizdeleong@gmail.com (G.R.-d.-L.); corteseslavadaniela@gmail.com (D.C.-E.); estherhp@us.es (E.H.-P.); maserrera@us.es (M.-Á.S.-F.)

**Keywords:** biopolymers, facial aesthetics, dermal fillers, gel-based systems, facial rejuvenation, hydrogels, biocompatibility, complications

## Abstract

Biopolymer-based dermal fillers have gained attention in facial aesthetics due to their biocompatibility, gel-forming properties, and capacity to stimulate tissue regeneration. However, evidence regarding their clinical performance remains scattered and inconsistent. This systematic review evaluates the current scientific literature on the effectiveness and safety of injectable biopolymers used in facial aesthetic procedures. A systematic search was conducted in PubMed, MEDLINE, and Embase databases for studies published between 2016 and 2024. Only human studies in English assessing clinical efficacy, safety, adverse events, and patient satisfaction were included. Of the 280 articles initially identified, 9 met the inclusion criteria. The selected studies showed improvements in facial volume and wrinkle reduction with gel-based biopolymers such as poly-L-lactic acid (PLLA), polycaprolactone (PCL), and polymethylmethacrylate (PMMA). Most studies reported high patient satisfaction and a low incidence of serious adverse effects. However, methodological heterogeneity and limited long-term data reduced the strength of the evidence. While injectable biopolymers appear to be effective and generally safe, current evidence is limited and variable. Further multicenter randomized trials with standardized protocols and longer follow-up periods are needed. Clinicians should apply these materials with caution, ensuring individualized treatment planning and careful risk assessment.

## 1. Introduction

Facial aging is a complex process involving various genetic, biological, and environmental factors [1,2,3,4]. This process is characterized by an unbalanced distribution of soft tissues, alterations in skin texture and tone, and wrinkles and folds [5]. In addition, it has been linked to several histological and molecular alterations, including a decrease in physiological hyaluronic acid (HA), dermal collagen, and elastin, which causes a gradual thinning of the skin and a reduction in elasticity [5,6].

Many of the biopolymers currently used in facial aesthetic applications—such as poly-L-lactic acid (PLLA), polycaprolactone (PCL), and polymethylmethacrylate (PMMA)—exhibit gel-like properties or are formulated into hydrogel systems for injection. These gel-based materials are favored for their ability to mimic the extracellular matrix, offer tunable mechanical properties, and ensure controlled degradation and integration into the host tissue. Hydrogels formed from these biopolymers provide an ideal medium for soft tissue augmentation, volumization, and sustained dermal stimulation, which explains their widespread use in facial rejuvenation protocols.

Dermal fillers have revolutionized the field of facial aesthetics, offering nonsurgical alternatives for rejuvenation and correction of skin imperfections [7]. These procedures range from removing wrinkles and fine lines to restoring volume and redefinition of facial contours [8]. Using a variety of biocompatible materials, dermal fillers provide temporary or permanent aesthetic improvements, establishing themselves as a prominent option in anti-aging strategies [9,10].

Socially, the aim of this type of treatment is to maintain appearance and expressiveness, so taking these aspects of the subject into account before treatment is fundamental to obtaining a natural result [11]. Implicitly, natural-looking results reflect universal notions of beauty while being individualized based on facial appearance and movement. A study by Nowell Solish et al. in 2019 [12] explored techniques and evaluation criteria to assess the naturalness of facial movement and expression following optimal bilateral correction of moderate to severe nasolabial folds and marionette lines with soft tissue hyaluronic acid fillers formulated with XpresHAn Technology™ resulting in lower facial naturalness with static expressions in full contraction “not adversely affected” compared to baseline for 100% of subjects during neutral, open smile, and grimace expressions. In the case of closed smiles and lip-pursing expressions, naturalness was not negatively affected in 96.7% of cases [12].

Despite its popularity and proven efficacy, applying dermal fillers is not without risk. Rohrich et al. [13] described complications as early (<14 days), late (14 days–1 year), and delayed (>1 year). Although most complications are mild and transient [14], complications such as arterial embolization, granuloma formation, and adverse reactions have been observed [14,15,16]. These adverse events underscore the need for a thorough understanding of the safety and efficacy of these treatments [17].

**Figure 1:**
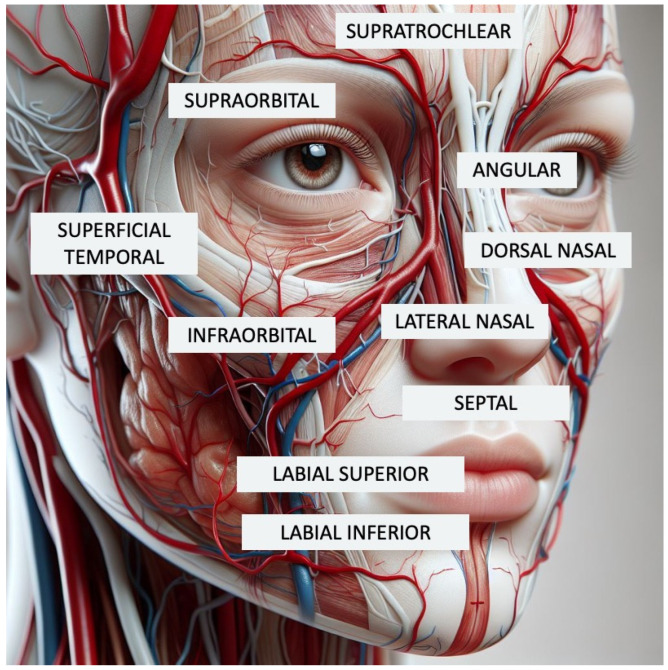
Facial vascular anatomy overlayed on a human face, illustrating the superficial arterial network commonly involved in aesthetic procedures. The image highlights key vascular regions such as the supratrochlear, supraorbital, angular, dorsal nasal, lateral nasal, infraorbital, superficial temporal, labial superior, and labial inferior arteries, providing visual context for injection safety in facial biopolymer applications.

A thorough knowledge of facial anatomy is a key factor in preventing complications [15]. Particularly, high-risk areas for vascular complications include the glabella, temporal fossa, lacrimal groove, midface, nasolabial folds, and nasolabial dorsum due to the large vessels in these areas (Figure 1) [18,19].

The growing demand for noninvasive aesthetic procedures, mainly dermal fillers, has prompted a comprehensive review of the available literature to assess the uniformity and quality of studies in this field. This literature review includes nine studies analyzed to determine the meta-analysis’s feasibility. The analysis criteria cover the interventions applied, the populations studied, the results obtained, and the quality of the methodological design of each investigation. The diversity of studies and the depth of recent research contribute to consolidating existing knowledge and identifying critical areas for future research and development.

The main objective of this study is to evaluate the consistency and quality of the current literature on the use of dermal fillers in facial rejuvenation procedures to identify areas that require methodological improvements and greater demographic inclusion in future research. This analysis seeks to facilitate more robust and reliable meta-analyses that can serve as a basis for optimizing clinical practices in facial aesthetics. The aims of this study are as follows:

- Assess the homogeneity in interventions, populations, outcomes, and design quality of the reviewed studies, providing a clear view of the variability and limitations of the current data.
- Identify opportunities for standardizing protocols and measurements in future studies, which would enable more accurate comparisons and facilitate combined analyses.
- Expand the evidence base on dermal fillers to diverse demographics, thereby improving understanding of their effects and safety in various populations.
- Promote the implementation of rigorous methodological designs, including randomization and adequate controls, to improve the quality and validity of studies in this field.

This approach seeks to consolidate and expand knowledge about dermal fillers and optimize their clinical benefits, ensuring that they are based on the best available evidence.

## 2. Results and Discussion

### 2.1. Study Selection and Data Extraction

Initially, 280 scientific articles were identified. After applying specific filters related to the facial anatomic area, human treatment, and types of materials used, 77 articles were selected for further analysis. The final review of these studies was performed using a checklist that included the following key elements (Table 1) [20,21].

A specific clinical question: does the study address the clinical question about the efficacy and safety of dermal filler biopolymers in facial aesthetics?

**Figure 2:**
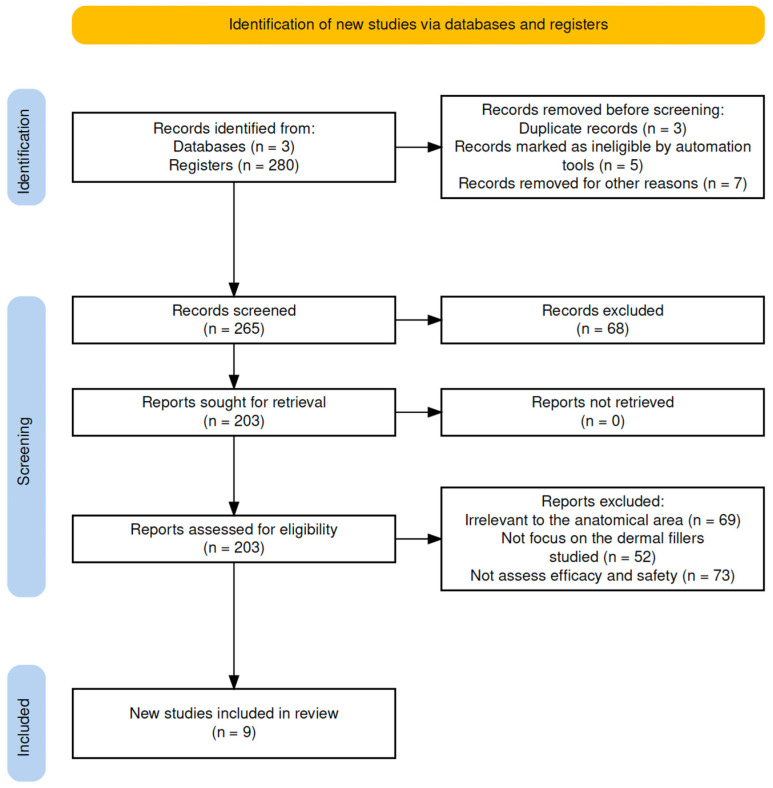
PRISMA 2020 flow diagram for new systematic reviews that included searches of databases.

In addition, CADIMA [2], a systematic review management tool developed by the Julius Kühn Institute, designed to enhance transparency and traceability in evidence synthesis, automatically generated a PRISMA [22] flowchart, documenting each stage of the review process from the initial search to the final inclusion of studies, thus ensuring transparency and accuracy in our findings (Figure 2).

### 2.2. Main Search Results

The data extracted for each article are shown in Table 2. Table 2 provides a concise summary of the core findings reported in each of the included studies. For each reference, the study type, main outcomes, and authors’ conclusions are presented. This overview allows for a quick comparative evaluation of the evidence regarding the safety and efficacy of biopolymer-based dermal fillers.

More specifically, a table was designed based on the essential characteristics, such as study design, number of participants, type of intervention, and results obtained (Table 3).

This table outlines the methodological details of each included study, such as study design, number of participants, specific interventions applied, and observed effects. These parameters are crucial to assess the internal validity and clinical applicability of the results.

### 2.3. Quality Assessment and Risk of Bias

Risk of bias assessment and critical reading were carried out using CADIMA, which allowed the application of specific criteria for each type of study. The quality of the clinical studies was assessed using criteria such as randomized sequence to verify whether the assignment to the interventions was randomized using methods such as computer-generated numbers or random number tables; sequence concealment to confirm that both investigators and participants were unaware of the assignment before the start of the study; double blinding to ensure that neither investigators nor participants were aware of the assigned group during the conduct of the study; and description of dropouts to ensure that the analysis included all participants who dropped out of the research or whose follow-up was lost, detailing the reasons for dropping out by group (Table 4).

### 2.4. Variety in Study Design and Levels of Evidence

The studies reviewed present considerable variation in their designs, ranging from retrospective and prospective reviews to case series to randomized controlled clinical trials. Levels of evidence also vary, with some studies providing high-quality data through controlled clinical trials (level I–II), while others are based on expert opinion or case reports (level III–V). Methodological quality tends to be lower in case reports or retrospective studies. The strength of evidence is generally moderate to high in most studies, although some reviews or case reports show low strength (Table 5).

Table 5 classifies the studies according to their methodological design, level of evidence, and strength of findings. This categorization is essential for determining the reliability of the studies and identifying which findings can be generalized to clinical practice.

The evaluation of the strength of evidence suggests that, although some studies provide robust data with multicenter methodologies and long-term follow-up, others are limited by non-experimental or retrospective designs and the lack of control groups. These differences are crucial when applying the findings to clinical practice, and it is advisable to confirm the results in well-designed and controlled studies. The last two studies reviewed offer valuable data with a sound methodology, although the first one presents a higher level of evidence due to its randomized clinical trial (RCT) design. It is important to consider these results in the context of their respective designs and to be aware of potential publication bias, especially in industry-funded studies, which may be biased toward positive results.

### 2.5. Heterogeneity Analysis

The included studies exhibit substantial heterogeneity across four main dimensions: study design, population characteristics, type of intervention, and reported outcomes. This variability has important implications for the interpretation and comparability of the data.

In terms of study design, we observed a mixture of randomized clinical trials, prospective cohorts, retrospective reviews, case series, and descriptive analyses. This affects both the internal validity and the strength of the evidence available.

The populations studied ranged from general adult patients to specific ethnic groups (e.g., Chinese women) with differing aesthetic needs and risk profiles. This diversity limits the generalizability of findings to broader patient groups.

Regarding interventions, the studies analyzed various gel-based biopolymers—such as PLLA, PCL, and PMMA—as well as alternative techniques like thread lifts or laser treatments. These approaches are inherently different in terms of mechanism, application, and intended outcomes.

Lastly, the outcome measures varied significantly, including subjective assessments (e.g., patient satisfaction, visual improvement scales), objective markers (e.g., wrinkle scores, skin histology), and complication rates. The lack of standardized outcome metrics further complicates direct comparison across studies.

Table 6 below summarizes this heterogeneity and illustrates why data pooling is not appropriate. Instead, this diversity highlights the need for greater methodological standardization in future clinical studies on biopolymer fillers.

Table 6 presents a summary of heterogeneity across design, population, intervention, and outcomes in the reviewed studies.

### 2.6. Recommendation

To assess the feasibility of meta-analysis and subgroup meta-analysis based on the 9 articles provided, it is crucial to consider several factors, including the homogeneity of study populations, interventions, and outcome measures, as well as the quality and design of the studies.

In the Homogeneity of Interventions and Populations study, studies vary significantly in terms of interventions, from dermal filler injections to threading and laser techniques, complicating direct comparability. Study populations also vary (adults with wrinkles, Chinese women specifically, patients with nasolabial folds, etc.), which adds another layer of heterogeneity.

Outcome measures differ among studies, ranging from subjective aesthetic improvements to objectively measured outcomes such as epidermal thickness and collagen formation. The quality and design of studies range from randomized controlled trials to case series and methodological reviews, affecting the uniformity in the quality of evidence (Table 7).

Table 7 evaluates the feasibility of conducting a global or subgroup meta-analysis based on the homogeneity of interventions, study populations, outcomes, and methodological quality. This assessment provides insight into the appropriateness of data synthesis and identifies areas requiring greater standardization.

**Figure 3:**
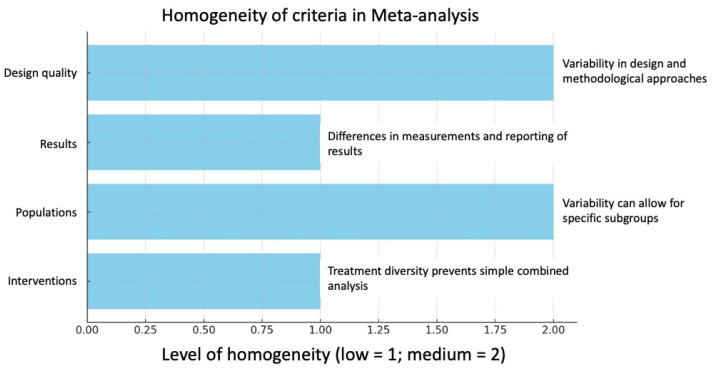
Homogeneity of criteria graph for meta-analysis. The graph provides a clear visualization of areas that could benefit from further standardization or more uniform methodological approaches in future studies.

A global meta-analysis is not recommended due to the high heterogeneity observed in the studies. However, performing subgroup meta-analyses could be feasible and useful to explore specific effects in more homogeneous populations or interventions, allowing a more accurate and relevant interpretation of the available data (Figure 3).

The horizontal bar chart (Figure 3) shows the different criteria evaluated in the study with their respective levels of homogeneity and comments associated with each one:

- Interventions low homogeneity due to the diversity of treatments, which precludes a simple combined analysis.
- Populations show average homogeneity, and the observed variability could allow the identification of specific subgroups.
- Results also have low homogeneity, with notable differences in measurements and reporting of results.
- Design quality shows average homogeneity with variability in the designs and methodological approaches used.

### 2.7. Effectiveness and Safety Evaluation

It is evident that most articles do not provide sufficient data to directly calculate the risk difference, the number needed to treat (NNT), or the number needed to harm (NNH). This is mainly due to the lack of comparative control groups or the absence of specific pre- and post-treatment incidence data.

Despite the lack of rigorous quantitative data, several studies report high levels of patient satisfaction and a low incidence of serious adverse events, suggesting a general perception of effectiveness and safety. For example, the article on treatment with Sculptra Injectable indicates significant effectiveness, with noticeable improvements in cheek wrinkles after approximately three treatments, albeit with an NNH of 5, noting that adverse events are not uncommon.

For all studies, the risk difference, NNT, and NNH are categorized as “Not directly calculable,” underscoring the need for future studies with more robust methodological designs that include controlled comparisons. The absence of control groups and lack of specific data significantly limit the ability to rigorously assess the safety and effectiveness of the investigated treatments. This highlights the importance of designing prospective studies and controlled clinical trials to obtain more accurate and reliable assessments.

Of the nine studies evaluated, only one provided sufficient data for a complete analysis of RD, NNT and NNH. This study highlighted an NNT of approximately 3 and an NNH of 25 for polycaprolactone-based fillers, indicating high efficacy and moderate risk of adverse effects. The remaining studies provided qualitative results or insufficient data for accurate calculations, underscoring the need for standardization in reporting clinical outcomes. Although the article provides qualitative efficacy and safety data, it does not provide specific incidences of adverse effects compared to a control or other treatment group, which is necessary to calculate the risk difference between NNT and NNH. However, given that it reports sustained improvement with no serious adverse events, we can infer that the treatment was well tolerated and effective.

The application of risk difference, NNT, and NNH could only be performed in the article evaluating the effectiveness and safety of Sculptra Injectable (PLLA-SCA) (Table 8).

The gel-forming nature of many injectable biopolymers—such as PLLA and PCL—plays a crucial role in their clinical behavior. As hydrogels, these materials create a scaffolding network that not only fills volume but also supports cellular infiltration and neocollagenesis. Their controlled degradation kinetics and moldability make them suitable for personalized treatments in aesthetic medicine. The reviewed studies support the effectiveness of these gel-based formulations in providing sustained aesthetic improvements, with a generally favorable safety profile when appropriate techniques are used.

This table summarizes the application of key clinical effect measures—including risk difference (RD), number needed to treat (NNT), and number needed to harm (NNH)—for the subset of studies where such calculations were feasible. These indicators provide a quantitative understanding of treatment efficacy and safety.

### 2.8. Baseline and Relative Risk Assessment

Given the type of study, baseline and relative risk assessment is only applicable in clinical trials and prospective studies, where direct comparison of interventions allows a clear assessment of treatment effectiveness [23,24,25]. In other types of studies, such as reviews and case studies, it is not possible to assess baseline and relative risk due to the lack of a comparative control group. Levels of publication bias vary significantly. Articles based on reviews, both retrospective and literature reviews, have a high risk of publication bias, mainly because such studies may prefer to publish positive or more striking results [26,27,28,29]. Case studies show a very high risk of bias, possibly due to the selection of cases highlighting particular outcomes [30,31]; in contrast, clinical trials and prospective studies exhibit a moderate risk, reflecting a more balanced and controlled approach that includes the publication of results regardless of their positive or negative nature [23,24,25].

Baseline risk, relative risk, and publication bias were also assessed for the nine articles mentioned, based on their study types (Table 9).

The table underscores the importance of selecting the appropriate study type to address specific research questions and minimize publication bias. For evaluations of treatments such as dermal fillers, clinical trials and prospective studies provide more reliable and comparable data, whereas reviews and case studies should be interpreted with caution because of their potential for high publication bias.

### 2.9. Discussion

Based on the exhaustive analysis of the articles reviewed and the considerations discussed previously, we note that the results of the current studies on dermal fillers and biopolymers should be interpreted with caution. The reliability of these results is affected by low homogeneity in interventions, populations studied, and methods of outcome measurement, indicating significant variability. This suggests that, although the studies provide valuable insights, they may not be fully representative or generalizable without additional considerations.

Studies on biopolymers such as PLLA and PCL for aesthetic treatments exhibit considerable variability in terms of methodological design, ranging from randomized clinical trials to case series and reviews, with levels of evidence ranging from I to V. This methodological diversity underscores the difficulty of directly comparing results and assessing the consistency of reported effects. The research by Kim et al. in KoreaMed and the analyses by Signori et al. in MDPI reflect this variability, indicating both promising results and the need for cautious interpretation of the data due to the observed heterogeneity [32,33].

Recognizing and managing heterogeneity in studies, as well as the impact of publication bias on the interpretation of research results in aesthetic medicine, is important. Methodological variability between studies can significantly affect conclusions about the effectiveness and safety of interventions, and publication bias can distort the overall perception of treatment efficacy. This information is crucial to critically evaluate the existing literature and to design future research to mitigate these problems [34].

Retrospective studies and reviews lack comparative control groups, which limits their ability to assess baseline and relative risk [26,27,28,29]. These studies rely heavily on historical data and are inherently subject to high publication bias due to the possible selection of cases or studies that support desired outcomes. Despite their usefulness in generating hypotheses, these approaches do not provide the robustness needed to confirm the safety or effectiveness of treatments.

Although retrospective studies can provide useful information to formulate hypotheses, they lack the rigorous controls necessary to draw definitive conclusions about the safety and effectiveness of treatments. In addition, the lack of comparative control groups limits their ability to assess baseline and relative risk, preventing accurate and robust interpretation of the results [35].

On the other hand, clinical trials and prospective studies apply methodologies that allow direct and controlled comparisons between treatments and controls [23,24,25]. These studies can accurately assess baseline and relative risk, providing a stronger basis for statistically valid conclusions about the effectiveness and safety of treatments. In addition, the moderate risk of publication bias reflects a commitment to transparency and scientific integrity.

Publication bias is especially prominent in case studies [30,31], where the selection of interesting or successful cases may distort the perception of treatment effectiveness. This bias not only affects the interpretation of individual results but may also influence the overall literature, promoting a biased positive view of certain interventions [36].

Publication bias can significantly influence the medical literature, highlighting how positive results are more likely to be published than negative or neutral ones, which can distort the available evidence on the effectiveness and safety of medical interventions, including those in aesthetic medicine [37,38].

The variability in the quality and rigor of the studies reviewed underscores the need for critical evaluation of the literature before applying findings to clinical practice. Clinicians should be aware of the limitations of less rigorous studies and prefer those with more robust methodologies to inform their therapeutic decisions.

The existing evidence on the effectiveness and safety of dermal fillers and biopolymers is not as strong as would be desirable to support unqualified standard clinical practice. The evidence is limited by diversity in study design, lack of rigor in methodology, and limited duration of follow-up. The need for more rigorous and standardized studies is clear to strengthen the evidence base [39].

The results suggest that while some treatments such as Sculptra Injectable show significant effectiveness, they also present a risk profile that should not be ignored. The need for approximately three treatments for a beneficial outcome, along with an NNH of 5, indicates that adverse events are not uncommon and should be considered when assessing the benefit–risk balance of treatment [23].

The analysis of effective strategies to minimize adverse events in aesthetic treatments with poly-L-l-lactic acid (PLLA) highlights the importance of addressing common reactions such as papules, nodules and, in rare cases, granulomas, typically associated with PLLA injection. The relevance of employing precise injection techniques, carefully selecting patients, and properly managing their expectations to reduce the incidence of complications is emphasized. Most adverse events are controllable and can be mitigated by a meticulous and considerate approach. This information is crucial for practitioners applying these treatments, as it contributes to improving safety in aesthetic interventions, ensuring safer and more satisfactory outcomes for patients [40].

To generate reliable recommendations for future clinical practice, it is crucial to encourage more randomized clinical trials and prospective multicenter studies that include diverse demographic groups. These studies should strive to minimize publication bias by pre-registration of trials and mandatory publication of all results, positive or negative. In addition, it would be beneficial to standardize outcome measurement methods and assessment criteria to improve comparability between studies.

Future research should employ various satisfaction scales, and it will be better to take into account the time frame due to the temporal effect of the fillers. Evaluation of patient satisfaction at different time points (1, 3, 6, and 12 months) will elucidate the duration of the procedure [41].

Among the most important clinical considerations, the need for accurate diagnosis of adverse reactions by biopsies and histopathological studies stands out, which is essential for proper and timely management. It should be emphasized that correct identification of the filler material involved and understanding of the tissue response are crucial for choosing the correct treatment and preventing future complications [42].

In conjunction with these findings, it is important to highlight that many of the biopolymers analyzed in this review are used in the form of gel-based systems or injectable hydrogels, which offer significant clinical advantages. These gels not only provide mechanical support and immediate volumization but also act as bioactive matrices that promote tissue regeneration and progressive collagen stimulation. Their three-dimensional structure, high water-retention capacity, and biocompatibility are key characteristics that contribute to their success in minimally invasive aesthetic procedures. This gel-based approach should be considered a research priority, particularly in the development of safer, longer-lasting formulations tailored to different facial phenotypes.

## 3. Conclusions

This systematic review presents the first focused synthesis of gel-based injectable biopolymers used in facial aesthetics, specifically evaluating materials such as poly-L-lactic acid (PLLA), polycaprolactone (PCL), and polymethylmethacrylate (PMMA). While many of these products have been gradually replaced in some markets by newer resorbable fillers like hyaluronic acid, they remain in clinical use and are relevant to both current practice and regulatory oversight.

Our findings indicate that biopolymer fillers formulated as gels offer promising outcomes in terms of volumization, collagen stimulation, and overall patient satisfaction. However, the body of evidence supporting their safety and efficacy remains fragmented and methodologically heterogeneous. Most studies included in this review exhibit limitations such as small sample sizes, non-randomized designs, and inconsistent outcome measures.

This review contributes novel insight by critically appraising the long-term behavior of these materials as hydrogels, emphasizing their role not only as volumetric agents but also as bioactive matrices. It also identifies a lack of standardized protocols and highlights the importance of rigorous study designs for evaluating adverse events, especially delayed complications such as granulomas or vascular compromise.

From a clinical perspective, these findings underscore the need for careful material selection, individualized patient planning, and informed consent that includes discussion of long-term risks. Practitioners should also be equipped to diagnose and manage filler-related complications, with support from histopathological tools when necessary.

Future research should prioritize multicenter randomized controlled trials with long-term follow-up, standardized aesthetic outcome measures, and comparative analyses against newer-generation fillers. Such efforts are essential to strengthening the evidence base and guiding the safe, effective, and evidence-based use of gel-forming biopolymer fillers in modern aesthetic medicine.

## 4. Materials and Methods

### 4.1. Protocol and Registration

The study protocol was prepared based on the Preferred Reporting Items for Systematic Review and Meta-Analysis (PRISMA) [37] statement, and the transparency of the review was increased using the PRISMA checklist. The developed protocol was previously registered and allocated the identification number CRD420251009829 in PROSPERO, the International Prospective Register of Systematic Reviews database, hosted by the National Institute for Health Research (NIHR), Centre for Reviews and Dissemination, University of York, York (UK) (www.crd.york.ac.uk/PROSPERO, accessed on 12 March 2025).

### 4.2. Focus Question

The focus question was designed according to the PICO question [2]: in patients undergoing facial aesthetic procedures, are biopolymers as dermal filler material more effective and safer than other dermal fillers or no treatment?

The PICO elements were as follows:

- P (Population): Patients undergoing facial aesthetic procedures with dermal fillers.
- I (Intervention): Use of biopolymers as dermal filler material.
- C (Comparison): Other dermal fillers (such as hyaluronic acid or calcium hydroxyapatite) or no treatment.
- (Outcome): Evaluation of efficacy (aesthetic improvement and patient satisfaction) and safety (presence of adverse effects and complications).

### 4.3. Search Strategy

The PubMed, MEDLINE, and Embase databases were searched for publications in peer-reviewed journals. Only publications written in English and published in the last eight years (2016–2024) were selected. Bibliographies of previously published systematic reviews, meta-analyses, and literature reviews were examined to identify relevant manuscripts not included in the electronic search. The following search strategy design was used for each database:

MEDLINE (via PubMed): (“Biopolymers”[MeSH] OR “Biocompatible Materials”[MeSH]) AND (“Dermal Fillers”[MeSH] OR “Cosmetic Techniques”[MeSH]) AND “Humans”[MeSH] AND (2016:2024[dp])

Embase (via PMC): (‘biopolymer’/exp OR ‘biocompatible material’/exp) AND (‘dermal filler’/exp OR ‘cosmetic procedure’/exp) AND ‘human’/exp AND (2016–2024)/py

### 4.4. Inclusion and Exclusion Criteria for Studies

The inclusion criteria for this study were defined to ensure the relevance and quality of the data collected. Only observational studies, clinical trials, systematic reviews, case studies, and meta-analyses that provided quantitative or qualitative data on the use and effectiveness of biopolymers and other fillers in facial aesthetics were included.

The selected studies had to have been performed in humans and published between 2016 and 2024 to ensure the timeliness of the information and its relevance in the context of the latest technologies and practices in aesthetic medicine. Only articles in which biopolymers were applied to the facial dermis or hypodermis were considered. In addition, only publications in English were included to cover a wide range of international research.

The types of fillers evaluated specifically included biopolymers, poly-L-lactic acid (PLLA), polydioxanone (PDO), and polymethylmethacrylate (PMMA). Priority was given to studies that reported results on efficacy, safety, complications, patient satisfaction, and comparisons with other treatments or materials, as well as those that provided clinically relevant information for facial rejuvenation and correction (Table 10).

### 4.5. Selection Process and Data Collection

The electronic and manual literature search was conducted using CADIMA as a central tool to manage and document the systematic review process. Two independent reviewers (G.R.-d.-L and D.C.-E.) performed the selection of studies applying predefined inclusion and exclusion criteria, ensuring a rigorous and transparent selection of the most relevant studies. CADIMA facilitated the critical assessment of study quality using a scoring scale based on study design, sample size, standardized instruments, and participant selection methods.

Full-text articles were independently reviewed by both investigators to determine their eligibility. Any discrepancies in the inclusion of studies were discussed until a consensus was reached, with the participation of a third reviewer (M.B.-D.) when necessary. Studies that did not meet the eligibility criteria were excluded, and the reasons for exclusion were formally recorded. CADIMA was used as a central tool to manage and document the systematic review process in this systematic review. CADIMA allowed us to rigorously define and apply inclusion and exclusion criteria, facilitating the transparent selection of the most relevant studies.

Critical reading and risk of bias assessment were performed using CADIMA, which facilitates quality assessment of the study using tools adapted to the design and methodology of each type of study. CADIMA provides functionalities to apply specific quality and risk of bias criteria for different studies, including randomized, cohort, case–control, and case series. Data extraction and assessment of the internal validity of the studies were also managed through this tool, ensuring a structured and transparent analysis. In the last selection phase, the quality of the clinical studies was assessed using the following criteria: random sequence to verify whether the allocation of patients to interventions was genuinely random, employing methods such as computer-generated numbers or random number tables; sequence concealment to confirm whether robust procedures were applied to ensure that both investigators and participants were unaware of the allocation before initiating the study; double blinding to ensure that neither investigators nor participants were aware of the assigned group during the conduct of the study; and description of dropouts, ensuring that the analysis included all participants who dropped out or whose follow-up was lost, detailing numerical data and reasons for dropping out in each group.

Three investigators performed data extraction in duplicate to ensure accuracy (E.H.-P.; M.-A.S.-F. and D.T.-L.). The data extracted included the authors and year of publication, study design, sample size, type of biopolymer used, anatomic area treated, and results in terms of efficacy, safety, complications, and patient satisfaction.

In addition, CADIMA automatically generated a PRISMA flowchart, documenting each stage of the review process from the initial search to the final inclusion of the studies.

## Figures and Tables

**Table 1:** Search thread for all databases.

Search Strategies	Number of Studies Available
A number of studies were found using the search terms “BioPolymers,” Aesthetic Facial,” and “Humans.”	280
The total number of studies excluded was based on eligibility criteria.	203
A total number of studies was excluded because they were review articles or did not provide complete articles.	62
Total number of studies accepted and reviewed	9

**Table 2:** Main search results.

Article	Authors	Type of Study	Main Results	Conclusion	Summary
Forehead Contouring with a Polycaprolactone-Based Dermal Filler [23]	Bae B, Lee G, Oh S, Hong K	Clinical trial	Favorable and long-lasting cosmetic results for forehead augmentation with PCL	PCL is safe and effective for forehead contouring	Evaluation of the long-term safety and efficacy of forehead contouring with a PCL-based dermal filler.
Biostimulatory Activity of Poly-L-Lactic Acid [24]	Arruda S, Prieto V, Shea C, Swearingen A, Elmadany Z, Sadick NS	Clinical trial	Significant improvement in skin quality with repeated PLLA treatments	PLLA improves skin quality through tissue remodeling	Clinical study evaluating biostimulant activity and longevity of PLLA for facial rejuvenation
Long-Term Safety of Polycaprolactone Filler [25]	Moers-Carpi M, Christen MO, Delmar H, Brun P, Bodokh I, Kestemont P	Prospective multicenter study	Long-term efficacy and safety of PCL filler in the correction of nasolabial folds.	Confirmation of the long-term safety and efficacy of PCL filler.	A prospective study evaluating PCL filler’s long-term safety and efficacy in correcting nasolabial folds.
Immediate Reconstitution of Poly-lactic Acid [26]	Vasconcelos-Berg R, Real J, Wenz F, Avelar LET	Multicenter retrospective study	Safety profile similar to that of pre-reconstituted PLLA	Safety of immediately reconstituted PLLA in aesthetic treatments	Safety evaluation of immediately reconstituted PLLA for facial and body treatments.
Synthetic Fillers for Facial Rejuvenation [27]	Lee JC, Lorenc ZP	Review	Efficacy in terms of volume and biological stimulation, safety in the use of synthetic fillers	Synthetic fillers are safe and effective for facial rejuvenation	Review of the use of synthetic fillers for facial rejuvenation
Safety of Injectable PLLA Used with Alternative Reconstitution [28]	Palm M, Mayoral F, Rajani A, Goldman MP, Fabi S, Espinoza L, Andriopoulos B, Harper J	Retrospective review	Low incidence of adverse events with higher reconstitution volumes	Confirmed safety with higher reconstitution volumes	Review of the safety of increased PLLA reconstitution volumes for facial treatments.
Facial Rejuvenation with Polydioxanone Thread [29]	Lee H, Yoon K, Lee M	Retrospective review	High patient satisfaction and favorable aesthetic results with polydioxanone yarns	Polydioxanone thread method safe and adequate for Asians	Review of the results of facial rejuvenation with polydioxanone threads for Asians.
Permanent Dermal Filler (Artecoll-4) in Chinese Women [30]	Li D, Luo SK, Wang YC, Lemperle G	Case Study	Safety and efficacy of large volumes of Artecoll-4 in Chinese women.	Artecoll-4 is safe and effective for facial volume restoration.	Case study on facial volume restoration with Artecoll-4 in Chinese women.
PMMA-Induced Nodules of the Lips [31]	Goldman A, Wollina U	Case review	Efficacy of the neodymium: YAG laser in the resolution of PMMA nodulations.	Laser techniques are effective in managing complications with PMMA	Review of PMMA lip complications and their management with lasers

**Table 3:** Specific characteristics of the studies analyzed.

Article	Study Design	Participants	Intervention/Exhibition	Links	Results (Estimated Effects)
Forehead Contouring with a Polycaprolactone-Based Dermal Filler [23]	Multicenter, retrospective	10,725 patients in China	Use of Artecoll-4 for facial volume restoration	Volumetric restoration and facial esthetic enhancement	Successful use with high satisfaction rates and long-lasting effects
Biostimulatory Activity of Poly-L-Lactic Acid [24]	Prospective, European multicenter	90 subjects with moderate/severe nasolabial folds	Polycaprolactone-based dermal filler	Long-term safety and efficacy	High aesthetic improvement sustained for up to 18 months
Long-Term Safety of Polycaprolactone Filler [25]	European multicenter, prospective	90 subjects with moderate/severe nasolabial folds	Polycaprolactone-based dermal filler	Safety and efficacy evaluation	84% showed improvement ≥ 1 point in WSRS at 12 months, with results maintained at 18 months.
Immediate Reconstitution of Poly-lactic Acid [26]	Systematic review	-	-	-	Synthesizing the results of systematic reviews on medical interventions.
Synthetic Fillers for Facial Rejuvenation [27]	Prospective clinical evaluation	58 patients (1 male, 57 females)	Polycaprolactone-based dermal filler for forehead augmentation	Efficacy evaluation using the GAIS scale	Positive results maintained up to 24 months post-treatment
Safety of Injectable PLLA Used with Alternative Reconstitution [28]	Randomized, controlled without treatment	Adults with moderate/severe cheek wrinkling	Poly-L-l-lactic acid injections (PLLA-SCA) vs. no treatment	Cheek wrinkle scale improvement	Significant wrinkle improvement at 7, 9, and 12 months with high patient satisfaction.
Facial Rejuvenation with Polydioxanone Thread [29]	Case report and review	-	Treatment of PMMA-induced labial nodules with neodymium: YAG lasers	Improvement of nodules and associated symptoms	Effective in the management of PMMA complications on lips
Permanent Dermal Filler (Artecoll-4) in Chinese Women [30]	Retrospective review of medical records	35 Asian patients	Facial rejuvenation with polydioxanone thread	Facial improvement assessed by photographs and patient satisfaction	High patient satisfaction with low complication rates
PMMA-Induced Nodules of the Lips [31]	Randomized, double-masked, placebo-controlled	Seven subjects in the treatment group and three subjects in the control group	Poly-L-l-lactic acid (PLLA) injections for histological skin analysis	Assessment of histological changes and skin quality	Improvement in skin quality with histological evidence

**Table 4:** Internal validity of the studies analyzed.

Article	Random Sequence	Sequence Hiding	Double Blind	Description of Abandonments
Forehead Contouring with a Polycaprolactone-Based Dermal Filler [23]	Yes (Clinical trial)	Yes	Yes	Detailed, including numbers and reasons for abandonment
Biostimulatory Activity of Poly-L-Lactic Acid [24]	Yes (Clinical trial)	Yes	Yes	Includes complete analysis of all registered participants
Long-Term Safety of Polycaprolactone Filler [25]	Yes (Prospective study)	Yes	Yes	Detailed, with long-term follow-up and explanations of casualties
Immediate Reconstitution of Poly-lactic Acid [26]	No (Retrospective study)	No (Retrospective study)	No (Retrospective study)	Not available (not applicable to retrospective studies)
Synthetic Fillers for Facial Rejuvenation [27]	No (Review)	No (Review)	No (Review)	Not available (literature review)
Safety of Injectable PLLA Used with Alternative Reconstitution [28]	No (Retrospective review)	No (Retrospective review)	No (Retrospective review)	Not available (not applicable to retrospective reviews)
Facial Rejuvenation with Polydioxanone Thread [29]	No (Retrospective review)	No (Retrospective review)	No (Retrospective review)	Not available (not applicable to retrospective reviews)
Permanent Dermal Filler (Artecoll-4) in Chinese Women [30]	No (Case study)	No (Case study)	No (Case study)	Brief description of abandonments and lost follow-ups
PMMA-Induced Nodules of the Lips [31]	No (Case review)	No (Case review)	No (Case review)	Not available (case review, not clinical trial)

**Table 5:** Variability in study designs and level of evidence.

Article	Description of the Study	Design Type	Level of Evidence	Strength of Evidence
Forehead Contouring with a Polycaprolactone-Based Dermal Filler [22]	Efficacy of polycaprolactone fillers	Prospective cohort	II	High
Biostimulatory Activity of Poly-L-Lactic Acid [23]	Effects of poly-L-l-lactic acid	Controlled clinical trial	II	Moderate
Long-Term Safety of Polycaprolactone Filler [24]	Safety of polycaprolactone fillers	Prospective multicenter study	II	High
Immediate Reconstitution of Poly-lactic Acid [25]	Review of techniques in systematic reviews	Methodological review	I	Moderate
Synthetic Fillers for Facial Rejuvenation [26]	Comparison of synthetic fillers	Descriptive review	III	Download
Safety of Injectable PLLA Used with Alternative Reconstitution [27]	Effectiveness of Sculptra on cheeks	Randomized clinical trial	II	High
Facial Rejuvenation with Polydioxanone Thread [28]	PDO Thread Rejuvenation	Retrospective study	III	Moderate
Permanent Dermal Filler (Artecoll-4) in Chinese Women [29]	Use of Artecoll-4 in Chinese women	Case series	IV	Download
PMMA-Induced Nodules of the Lips [30]	Treatment of nodules by PMMA	Case report and review	IV	Download
Forehead Contouring with a Polycaprolactone-Based Dermal Filler [23]	Efficacy of polycaprolactone fillers	Prospective cohort	II	High
Biostimulatory Activity of Poly-L-Lactic Acid [24]	Effects of poly-L-l-lactic acid	Controlled clinical trial	II	Moderate
Long-Term Safety of Polycaprolactone Filler [25]	Safety of polycaprolactone fillers	Prospective multicenter study	II	High
Immediate Reconstitution of Poly-lactic Acid [26]	Review of techniques in systematic reviews	Methodological review	I	Moderate
Synthetic Fillers for Facial Rejuvenation [27]	Comparison of synthetic fillers	Descriptive review	III	Download
Safety of Injectable PLLA Used with Alternative Reconstitution [28]	Effectiveness of Sculptra on cheeks	Randomized clinical trial	II	High
Facial Rejuvenation with Polydioxanone Thread [29]	PDO Thread Rejuvenation	Retrospective study	III	Moderate
Permanent Dermal Filler (Artecoll-4) in Chinese Women [30]	Use of Artecoll-4 in Chinese women	Case series	IV	Download
PMMA-Induced Nodules of the Lips [31]	Treatment of nodules by PMMA	Case report and review	IV	Download

**Table 6:** Heterogeneity of studies in terms of design, population, intervention, and results.

Article	Study Design	Study Population	Intervention	Main Results
Forehead Contouring with a Polycaprolactone-Based Dermal Filler [23]	Prospective cohort	Patients with facial contouring needs	Use of polycaprolactone fillers	Long-term efficacy in improving facial contours
Biostimulatory Activity of Poly-L-Lactic Acid [24]	Controlled clinical trial	Patients undergoing facial aesthetic treatment	Poly-L-l-lactic acid injections	Improved skin quality and increased collagen production
Long-Term Safety of Polycaprolactone Filler [25]	Prospective multicenter study	Patients with nasolabial folds	Polycaprolactone fillers	Long-term safety and efficacy in nasolabial fold correction
Immediate Reconstitution of Poly-lactic Acid [26]	Methodological review	N/A	Literature review	Synthesis of methods in systematic reviews
Synthetic Fillers for Facial Rejuvenation [27]	Descriptive review	N/A	Comparison of synthetic dermal fillers	Efficacy and safety evaluation of different fillers
Safety of Injectable PLLA Used with Alternative Reconstitution [28]	Randomized clinical trial	Adults with moderate/severe wrinkles	Sculptra Injections	Significant reduction in cheek wrinkles
Facial Rejuvenation with Polydioxanone Thread [29]	Retrospective study	Asian patients	Use of polydioxanone threads for facelifts	High patient satisfaction with minimal complications
Permanent Dermal Filler (Artecoll-4) in Chinese Women [30]	Case series	Chinese women	Artecoll-4 injections	Effective restoration of facial volume
PMMA-Induced Nodules of the Lips [31]	Case report and review	Patients with labial nodules	Laser and surgical treatment of nodules	Effective resolution of PMMA-induced nodules

**Table 7:** Summary of the feasibility of meta-analysis and subgroup meta-analysis based on homogeneity of interventions, populations, results, and design quality.

Criteria	Homogeneity	Comments on Feasibility of Meta-Analysis
Interventions	Download	Diversity of treatments prevents simple combined analysis
Populations	Media	Variability may allow for specific subgroups
Results	Download	Differences in measurements and results reporting
Design Quality	Media	Variability in design and methodological approaches

**Table 8:** Application of risk difference, NNT, and NNH.

Result	Treatment Group (%)	Control Group (%)	DR	NNT	NNH
Cheek Wrinkle Improvement	71.6	26.1	45.5%	3	N/A
Adverse Events	20.6	Assumption 0	20.6%	N/A	5

**Table 9:** Methodology and bias assessments in nine studies on dermal fillers and aesthetic treatments, highlighting the diversity in study type and level of associated publication bias.

Article	Type of Study	Baseline and Relative Risk	Publication Bias
Chart Review Presenting Safety of Injectable PLLA Used with Alternative Reconstitution Volume for Facial Treatments	Retrospective review	Not applicable	High
Safety of the Immediate Reconstitution of Poly-lactic Acid for Facial and Body Treatment-A Multicenter Retrospective Study.	Retrospective study	Not applicable	High
Synthetic Fillers for Facial Rejuvenation	Review	Not applicable	High
Safety and Long-Term Efficacy of Forehead Contouring with a Polycaprolactone-Based Dermal Filler	Clinical trial	Applicable	Moderate
Facial Volume Restoration with Permanent Dermal Filler (Artecoll-4) in Chinese Women	Case study	Not applicable	Very high
Outcome of facial rejuvenation with polydioxanone thread for Asians	Retrospective review	Not applicable	High
Polymethylmethacrylate-induced nodules of the lips: Clinical presentation and management by intralesional neodymium:YAG laser therapy.	Case review	Not applicable	Very high
A Clinical Histology Study Evaluating the Biostimulatory Activity Longevity of Injectable Poly-L-L-Lactic Acid for Facial Rejuvenation	Clinical trial	Applicable	Moderate
European Multicenter Prospective Study Evaluating Long-Term Safety and Efficacy of the Polycaprolactone-Based Dermal Filler in Nasolabial Fold Correction	Prospective study	Applicable	Moderate

**Table 10:** Inclusion and exclusion criteria.

Inclusion Criteria	Exclusion Criteria
Qualitative and quantitative studies	Studies on solid biopolymers
Studies conducted in humans	Exclusion of Specific Substances: Hyaluronic acid and agarose.
Publication Period: 2016 to 2024	
Studies published in English	
Biopolymer is studied as a material for dermal filler.	
Studies focused on facial aesthetics.	
Studies analyzing efficacy and safety of biopolymers in facial aesthetics.	

## Data Availability

All data supporting the findings of this study are included within the manuscript. However, additional details or clarifications can be provided upon reasonable request. Interested researchers may contact the corresponding authors for further information.
